# Survey of bar-lounges and restaurants regarding compliance with the current smoke-free regulation in Thulamela Municipality, South Africa

**DOI:** 10.4102/phcfm.v8i2.927

**Published:** 2016-06-24

**Authors:** Thikolelwi R. Nemakhavhani, Henry A. Akinsola

**Affiliations:** 1 Department of Community Services, Vhembe District Municipality, South Africa; 2 Department of Public Health, University of Venda, South Africa

## Abstract

**Background:**

The *Tobacco Products Control Act*, No. 83 of 1993 was introduced in South Africa in 1993. Due to the shortfalls of the 1993 Act, the *Tobacco Products Control Amendment Act*, No. 12 of 1999 was passed in 1999. The regulation relating to smoking of tobacco products in public places was gazetted in 2000 and implemented in 2001.

**Setting:**

The setting for the study was all selected registered licensed bar-lounges including restaurants within the municipality.

**Purpose of the study:**

To examine compliance levels with the current smoke-free regulation at bar-lounges and restaurants in Thulamela Municipality.

**Objectives of the study:**

To observe whether structural alterations had been effected to accommodate smoking patrons. To observe whether tobacco-related litter was present in non-smoking areas and in the outdoor areas within 5 meters of windows and entry ways. To observe whether individuals or groups engaged in smoking in non-smoking areas.

**Methods:**

An observational survey was conducted to measure the level of compliance by lounges and restaurants in Thulamela Municipality. A convenience sampling method was used to sample 56 bar-lounges, including restaurants. Data were collected using an observation log. Collected data were analysed using SPSS 20.0.

**Results:**

The study findings noted low compliance with the legislation with only one establishment (1.8%) complying with the requirements of the legislation.

**Conclusion:**

The level of compliance with the legislation is in a very low state in Thulamela Municipality. Further research is needed to explore factors influencing non-compliance with the regulation.

## Introduction

The World Health Assembly, the governing body of the World Health Organization (WHO) since 1976, recommended that the governments of its member states should give serious consideration to legislative measures for control of tobacco epidemic. This initiative was later reaffirmed by introduction of WHO Frame work Convention on Tobacco Control, which urged its member states to ensure the protection from exposure to environmental tobacco smoke. Of note in the framework is Article 8, which stipulates that:

after parties have recognized and agreed that scientific evidence has unequivocally established that exposure to tobacco smoke causes death, disease and disability, each party shall adopt and implement in areas of existing national jurisdiction as determined by national law and actively promote at other jurisdictional levels the adoption and implementation of effective legislation, executives, administrative and/or other measures, providing for protection from exposure to tobacco smoke in indoor work places, public transport, indoor public place and, as appropriate, other public places.^1^

According to Eriksen, Mackay and Ross,^2^ nearly 20% of the world’s population smoke cigarettes. It is estimated that approximately 600 000 individuals died from second-hand smoke in 2011, and 75% of these deaths were among women and children. Additionally, global prevalence of tobacco smoking revealed that more than 10 million cigarettes are smoked every minute of every day around the world, and if the trend continues, smokers would have consumed 9 trillion cigarettes annually by 2025.^3^

More recently in May 2014, the WHO during the observation of the ‘World No Tobacco Day’ released the latest updated information on the impact of tobacco on the population and reported that tobacco consumption kills approximately six million users annually.^4^ Of serious concern is that more than five million tobacco-related deaths are caused by smoking, which is of a grave concern, and that more than 600 000 of those deaths occur as a result of non-smokers being exposed to second-hand smoke.

As an initiative to control the tobacco epidemic, South Africa introduced the *Tobacco Products Control Act* in 1993. The 1993 Act was not considered to be comprehensive enough and the *Tobacco Products Control Amendment Act* was passed in 1999. This Act and its related regulation, that is, R No. 975 regulation, relating to smoking of tobacco products in public places among other prohibitions, prohibits smoking in public places, which includes workplace, restaurants, bars and public transport areas. The regulation also stipulates penalties for transgressors of the law and specifies the maximum permissible levels of tar and nicotine. The regulation was implemented in 2001.^5,6^

Regarding public places, the regulation emphasises that buildings should be completely smoke-free buildings or designate a smoking area that does not exceed 25% of the total floor area. A solid partition should separate the smoking area from the rest of the public place and an entrance door on which the sign ‘smoking area’ is displayed written in black letters, at least 2 cm in height and 1.5 cm in width, on a white board and ventilation of the designated smoking area should ensure that air from this area is directly exhausted to the outside and is not re-circulated to any other area within the public place.

Furthermore, the following message should be displayed:

smoking of tobacco products is harmful to your health and to the health of women and non-smokers. For help to quit phone (011) 7203 145 is displayed at the entrance to the designated smoking area, written in black letters, at least 2cm in height and 1.5 cm in breadth on a white background and notices and signs indicating the areas where smoking is permitted and where it is not permitted must be permanently displayed and signs indicating that smoking is not permitted must carry the warning: any person who fails to comply with this notice shall be prosecuted and may be liable to a fine.^6^

Tobacco growing and use does not affect population health only, but it has an adverse impact on the environment, economy and society at large. Worldwide, it is the poor who suffer the greatest burden from tobacco use. In some of the poorest households in many countries, 5% of income is spent on tobacco.^7^ Tobacco use robs resources for basic needs like food, education, shelter and health care. Suffering from tobacco-related disease prevents the breadwinner from active work, which in turn reduces household wages, inhibits the ability to provide for and educate the children and drives the family further into poverty.^7^

The main objective of the legislation is to restrict smoking in public places. The situation in Vhembe is not the case, as cigarettes sales occur everywhere, even among street vendors. The local bar-lounge and taverns are not strict in enforcing this legislation as they are only after profit.

With such comprehensive legislation, which bans smoking in public places, prohibits advertisements and sponsorship of cigarette and sales of cigarette to minor, one might have thought that the government was in control of the situation, but it appears that this has not been the case, because according to Steyn et al.,^8^ William et al.^9^ and Fong et al.,^10^ there is evidence of non-compliance in places of recreation as required by the legislation.

### Significance of the study

The results of the study are expected to contribute to the knowledge needed by the municipal government and the environmental health practitioners to monitor and enforce compliance, in order to identify the areas that are at high risk of tobacco smoking epidemic and whether the Act needs to be strengthened. According to the legislation, municipalities have the power, duty and obligation to enforce the legislation in their areas of jurisdiction. The importance of this is that each municipality will be able to evaluate itself irrespective of whether it is fulfilling its obligation. The outcome of the study might also serve as a stimulus to other researchers interested in the area of tobacco use to conduct similar studies in other municipalities in South Africa for the purpose of comparison and building a body of knowledge on the subject at a national level.

### Purpose of the study

The purpose of the study was to examine compliance levels of bar-lounges and restaurants with the current smoke-free regulation in Thulamela Municipality.

### Objectives of the study

To observe whether structural alterations had been effected to accommodate smoking patrons.

To observe whether tobacco-related litter was present in non-smoking areas, in the outdoor areas within 5 meters of windows, in entry ways including doors and in other outdoor non-smoking areas.

To observe whether individuals or groups are engaged in smoking in non-smoking areas.

## Research methods and design

### Study design

A quantitative paradigm was used in this research. The design was chosen as a guide to assemble the data in order to achieve the objectives of this study.

### Target population and sample

The target population for the study were selected registered licensed places of entertainment found within the 38 Thulamela municipal wards. As to the type of public places included in the study, the following formal establishments were targeted:

- Eating places, for example, restaurants, where the primary business is to sell food to the public.
- Drinking places, for example, local bar-lounges and registered taverns, where the primary business is to sell alcoholic beverages to the public.
- Combined drinking and eating places, for example, eating houses, where the primary business is to sell both alcoholic beverages and food to the public.
- The sampling was done in two stages in order to include licensed places of entertainment from both rural and semi-urban locations. In the first stage, the targeted 38 wards in the municipal area were classified according to whether they were rural or semi-urban for the purpose of comparison. In the second stage, of those classified as rural, four wards (21, 22, 26 and 27) were selected by balloting, among those classified as semi-urban, four wards (19, 20, 24 and 25) were selected by balloting; Central Business Districts were also randomly selected by balloting. Within each ward, there are a large number of licensed places of entertainment. However, because of limited human and financial resources, a convenient sample of 56 bar-lounges including restaurants were selected based on the inclusion and exclusion criteria.

Table 1 shows the public places included in the study and the results of the observation.

**TABLE 1:** Sampled licensed establishments observed (*N* = 56).

Serial number	Total number of establishments observed	Establishment category	Establishment type	Separate seating arrangements (designated smoking area)	

				Yes	No
1	23	Eating places	Restaurants	x	-
2	26	Drinking places	Bar-lounges and taverns	-	x
3	7	Combined eating and drinking places	Eating houses	-	x
**Total**				**1**	**55**

*Source:* Authors’ own work

### Inclusion and exclusion criteria

All registered licensed places of entertainment in Thulamela Municipality were included and all unregistered or unlicensed places of entertainment were excluded for control purposes.

### Research instrument

An observation log was used to observe and collect data to assess whether structural changes were effected in places of recreation and whether individuals or groups were engaged in smoking in ‘non-smoking areas’ in places of entertainment/recreation in the municipality. The observation log was adapted from previous instruments: The Utah Second Hand Policy Implementation Guide revised in January 2007 by Utah Department of Health, Tobacco Prevention and Control programme (USA) and the instrument used by the University of Free State Centre for Health Systems Research and Development for their study on the levels of compliance in public places with the current tobacco legislation. Because the instrument was used to gather information by the researchers through observation, there was no need to translate the instrument into the local language.^8,11^

### Data collection

The instrument was administered by a team comprising the two researchers. For each of the selected places of entertainment, a copy of the data sheet was completed by a team member in addition to recording direct observations in the observational log.

### Data analysis

The data were analysed using Statistical Package for Social Sciences (SPSS) 20.0 version. The results were presented in frequencies and percentages/proportions.

### Reliability and validity

The researchers did an extensive literature search on the adapted instrument and liaised with experts in the field of public health to ensure the validity and reliability of the instrument. To ensure reliability, the instrument was administered twice on two wards, which did not form part of the final study, and in five places of entertainment, which also did not form part of the units selected for the study. The instrument was then amended based on this field experience. The validity of the instrument was ascertained by giving it to experts in Public Health to review and make suggestions.

### Ethical consideration

The research proposal was submitted to the research ethics committees of the University of Venda for Science and Technology for approval (project No SHS: 9814249). Thereafter, it was submitted to Thulamela Municipality for approval before conducting the study. A consent form was signed by the owner’s or a representative of all places of entertainment included in the study. Furthermore, the permission letter to conduct the research from the Municipal manager was read to the owners of the establishments and voluntary participation was emphasised before any observation took place. The standard ethical consideration of anonymity and confidentiality was maintained throughout the research period.

## Results

### Results relating to structural alterations/changes

As depicted in Table 2, it was observed that the majority of sampled establishments (98.2%) did not undertake structural alterations in efforts to comply with the tobacco regulations. The predominant structural changes undertaken by only one establishment consisted of separate seating arrangements (1.8%), ensuring partition from floor to ceiling between non-smoking and smoking areas (1.8%), separate ventilation system which expelled the air to the outside of the smoking area (1.8%) and installation of a door (1.8%).

**TABLE 2:** Whether structural changes were made by the owners of the Licensed Places of Entertainment in Thulamela Municipality (*N* = 56).

Outcome of structural changes	Header					

	Yes		No		Total	
		
	*N*	%	*N*	%	*N*	%
Separate seating arrangements	1	1.8	55	98.2	56	100.0
Partition from floor to ceiling	1	1.8	55	98.2	56	100.0
Separate ventilation system	1	1.8	55	98.2	56	100.0
Installed a door	1	1.8	55	98.2	56	100.0
No changes at all (smoking is freely allowed)	40	71.4	16	28.6	56	100.0
No changes necessary (smoke-free establishment)	15	26.8	41	73.2	56	100.0
Smokers smoking among non-smokers	43	76.8	13	23.2	56	100.0

*Source:* Authors’ own work

Of the 56 sampled establishments, it was observed that in 40 (71.4%) places of recreation, there were no changes at all and smoking was freely allowed, and in 15 (26.8%) places of recreation, changes were not necessary as they were smoke-free establishments. The number of smokers who were found smoking among non-smokers was alarmingly very high at 43 (76.8%) (Table 2).

### Results relating to smoking practice

The majority of the establishments, 42 (70.0%), did not have designated indoor non-smoking areas; they were either places where smoking was freely allowed or smoke-free establishments. In 51 establishments (91.1%), tobacco-related litter was seen in outdoor areas within 5 meters of the windows, entry ways or doors. Furthermore, tobacco-related litter was seen in 50 (89.3) outdoor non-smoking areas, whereas 10.7% of the places of recreations’ outdoor non-smoking areas were free of any cigarette litter.

In 42 places of recreation (75.0%), people were seen either smoking or inhaling tobacco smoke in identified smoking areas, while only 2 patrons (3.6%) were not smoking. Twelve establishments (21.4%) had no designated smoking area and visitors were not seen smoking. In places of recreation where people were seen smoking, 21.3% were indoors, 10.6% were outdoors and 68.1% were seen both indoors and outdoors. Of the patrons observed, 6.4% were fewer than five in number and 93.6% were more than five. The majority of the smokers were visitors (95.7), while both employees and visitors who were smoking were seen in two establishments (4.3%). Table 3 shows the results of the observation related to smoking behaviour.

**TABLE 3:** Results of observation related to smoking practice.

Outcome of smoking observation	Variable	*N*	%
**Do you see tobacco-related litter, in non-smoking areas?**			
	Y	-	-
	N	14	25.0
	n/a	42	70.0
	**Total**	**56**	**100.0**
**Do you see tobacco-related litter in outdoor areas within 5 meters of windows, entry ways and doors?**			
	Y	50	89.3
	N	6	10.7
	n/a	-	-
	**Total**	**56**	**100.0**
**Do you see tobacco-related litter in other outdoor non-smoking areas?**			
	Y	50	89.3
	N	6	10.7
	n/a	-	-
	**Total**	**56**	**100.0**
**Do you see people smoking or inhaling tobacco smoke in smoking areas?**			
	Y	42	75.0
	N	2	3.6
	n/a	12	21.4
	**Total**	**56**	**100**
**Where do you see people smoking?**			
	Indoor	10	21.3
	Outdoor	5	10.6
	Both	32	68.1
	**Total**	**47**	**100.0**
**How many people do you see smoking?**			
	Less than 5	3	6.4
	More than 5	44	93.6
	**Total**	**47**	**100.0**
**Are the smoker’s employees, visitors or both**			
	Visitors	45	95.7
	Both	2	4.3
	**Total**	**47**	**100.0**

*Source*: Authors’ own work

## Discussion

The purpose of the *Tobacco Products Amendment Act* (No. 12 of 1999) and regulation made under it is to protect non-smokers from involuntary exposure to tobacco smoke by ensuring that establishments or places of recreation comply with the following conditions, that is, separate sitting arrangements with health and prosecution messages displayed at the entrance of the establishments, including observation of 5-meter distance away from the building entrances, windows and pathways while smoking in the outdoor areas. The South African legislation is guided by WHO resolutions and the WHO Framework Convention on Tobacco Control, which urges its member states to ensure protection from involuntary tobacco smoke.

The study results indicated 1.8% compliance in relation to the above-mentioned initiatives, as the majority of establishments (98.2%) did not implement structural changes in order to accommodate smokers. Similar results were noted in 2004 during the survey of restaurants regarding compliance with New York City smoke-free laws, where the levels of compliance ranged from medium, low to very low.^9^ In China, a survey conducted between 2007 and 2012 by Fong et al.^10^ found that as of 2012, out of 800 respondents, over three-quarters of respondents were exposed to smoking in bars, more than two-thirds were exposed to smoking in restaurants and more than half were exposed to smoking in indoor workplaces.

The unavailability of designated smoking area put the lives of no-smoking patrons in danger because of second-hand smoke. Tobacco-related litter (cigarette butts, empty cigarette packets) were not seen in indoor non-smoking areas at only 14 (25.0%) establishments. In 51 establishments (91.1%), tobacco-related litter was seen in outdoor areas within 5 meters of windows, entry ways or doors. Furthermore, tobacco-related litter was seen in 50 establishments in other outdoor non-smoking areas, whereas only 10.7% of outdoor non-smoking areas were free of any cigarette litter. This tobacco-related litter is evidence enough that our non-smoking patrons are not safe from environmental tobacco smoke. Similar findings were observed in many previous worldwide studies at large, and this has serious implications for health workers in their endeavour to reduce the spread of tobacco-related diseases, disability and death within Vhembe District.

A noteworthy study is the one titled ‘predicting smokers non-compliance with smoking restrictions in public places’. The aim of the study was to identify the predictors of non-compliance with smoking restrictions among Greek college student smokers. The study concluded that smoking was highly prevalent, and more than half of the current smokers reported not complying with existing regulations in public places. Smokers’ attitudes to smoking bans and tobacco control policies did not have an effect on compliance behaviour.^12^

The implication of non-adherence to the legislation in Thulamela Municipality should be a worrying factor for the authorities because it has been established that the impact of tobacco use is not limited to health issues only, but it is also a burden to the environment, economy and the society at large.

In the majority of the establishments (75.0%), people were seen smoking and the smell of tobacco smoke was identified in smoking areas. Patrons who were observed smoking who were fewer than five in number formed 6.4% while those who were more than five formed 93.6%. The high number of smokers observed confirms the findings on global prevalence of tobacco use, which revealed that more than 10 million cigarettes are smoked every minute of every day around the world, and by 2025, if the trend continues, smokers would have consumed 9 trillion cigarettes annually.^3^ Further studies shows that approximately 20.0% of the world’s population smoke cigarettes, including about 800 million men and 200 million women. It is estimated that 600 000 individuals died from second-hand smoke in 2011, and 75.0% of these deaths were among women and children.^2^

The majority of smokers observed in the study were visitors (95.7%) while smoking employees were seen in only two establishments. The high number of patrons who were observed smoking and the presence of tobacco-related litter everywhere raises a serious health issue, because it is already estimated that between 2020 and 2030, the number of tobacco-related deaths will increase to 10 million, with 7 million occurring in developing countries, including the African region.^3^

The non-adherence with the requirements of smoking policies and legislations poses serious health risks to non-smoking patrons because in June 27, 2006, the US Department of Health and Human Services (2006) released a major report titled *The Health Consequences of Involuntary Exposure to Tobacco Smoke: a Report of the Surgeon General*, which stated: ‘The SG report concluded that there is no risk-free level of exposure to second-hand smoke and that the only way to fully protect yourself and your loved ones from the dangers of second-hand smoke is through 100% smoke free environments’.^13^ WHO concurs with SG report according to recent statistics on tobacco-related deaths, indicating that tobacco use kills nearly 6 million people each year, with more than 5 million of the deaths occurring as a result of direct tobacco use, while 600 000 deaths are the result of non-smokers being exposed to second-hand smoke.^4^

### Limitations of the study

The major limitations of the study are that because of time factor the research was only able to cover a limited number and type of places of entertainment and informal or unlicensed establishments were not included in the study.

Secondly, it was not possible to interview the owners and/or managers of the places of entertainment included because it would have involved using two instruments instead of just the observation checklist; in other words, the researcher would have to also use a questionnaire. Therefore, the researchers advise the readers to be cautious in generalising the results of the study.

## Conclusion

Conclusions related to structural changes are as follows:

- Only one establishment undertook structural changes to comply with the Act.
- In the majority of the establishments, smokers were found smoking among non-smokers.
- No differences were found between semi-urban and rural settings with regard to compliance.

Conclusions related to smoking observation are as follows:

- The majority of the establishment had tobacco-related litter in both indoor and outdoor areas within 5 meters of windows, entry ways and doors because smokers do not observe a 5-meter distance from windows and entrances.
- The majority of the visitors were observed smoking in both indoor and outdoor areas. Some patrons even smoked in areas where smoking was not allowed, for example, non-smoking areas.

Generally, the article concludes that the compliance levels to the legislations with regard to smoking practices in public places are in very low state in Thulamela Municipality. An intervention to improve compliance is needed as a matter of urgency in order to remedy the current situation.
